# Transcriptomic signatures associated with underlying rapid changes in the early phase brain of bi-directional sex change in *Trimma okinawae*

**DOI:** 10.1098/rsos.231450

**Published:** 2023-12-06

**Authors:** Katsunori Tamagawa, Tomoki Sunobe, Takashi Makino, Masakado Kawata

**Affiliations:** ^1^ Graduate School of Life Sciences, Tohoku University, Aoba-ku, Sendai 980-8578, Japan; ^2^ Laboratory of Fish Behavioral Ecology, Tateyama Station, Field Science Center, Tokyo University of Marine Science and Technology, 670 Banda, Tateyama, Chiba 294-0308, Japan

**Keywords:** sequential hermaphroditism, sexual plasticity, brain, transcriptomics, *Trimma okinawae*

## Abstract

Teleost fish exhibit remarkable sexual plasticity and divergent developmental systems, including sequential hermaphroditism. One of the more fascinating models of sexual plasticity is socially controlled sex change, which is often observed in coral reef fish. The Okinawa rubble goby, *Trimma okinawae,* is a bi-directional sex-changing fish. It can rapidly change sex in either direction based on social circumstances. Although behavioural and neuroendocrine sex change occurs immediately and is believed to trigger gonadal changes, the underlying mechanisms remain poorly understood. In this study, we conducted a *de novo* transcriptome analysis of the *T. okinawae* brain and identified genes that are differentially expressed between the sexes and genes that were immediately controlled by social stimulation implicating sex change. Several genes showed concordant expression shifts regardless of the sex change direction and were associated with histone modification in nerve cells. These genes are known to function in the neuroendocrine control of reproduction in nerve cells. Overall, we identified genes associated with the initiation of sex change, which provides insight into the regulation of sex change and sexual plasticity.

## 1. Introduction

Sexual dimorphism is observed in a wide range of vertebrates. Females and males exhibit clear differences in behaviour, physiology and morphology [1,2]. In most species, the sexual fate of an individual is generally determined at an early phase of development [3,4]. Interestingly, teleosts exhibit divergent sex determination systems and retain extreme sexual plasticity throughout their life [5]. Various sex-determination systems, including environmental and genetic sex determination as well as sex change (i.e. sequential hermaphroditism), are observed in a diverse range of teleosts and their ecological and evolutionary roles have attracted significant interest [6].

One of the most fascinating models of sexual plasticity is sex change regulated by social status in coral reef fish [7,8]. Socially controlled sex change including protogyny (female-to-male), protandry (male-to-female) and bi-directional sex change (both directions serially) can occur rapidly [9]. In particular, behavioural sex change can occur rapidly and within an hour following a social status change and before gonadal change, whereas changes in the brain trigger the sex change in the fish [10,11]. Although gene expression change is likely to be involved in the initiation of sex change, transcriptional regulation in the brain during the early phase of sex change is poorly understood.

The Okinawa rubble goby, *Trimma okinawae,* can undergo bi-directional sex change. It is distributed between Kagoshima and southward to the Ryukyu Islands in Japan. A polygynous mating system occurs in *T. okinawae*, in which a dominant individual becomes male and exhibits territorial behaviour to maintain the harem, whereas the others are females developed from juveniles or changed their sex and exhibit subordinate behaviour [12]. The dominance hierarchy of *T. okinawae* is based on body size within the harem. The largest female changes its sex to male if males leave the harem; however, these males can change back into females upon encountering a larger, more dominant fish [12,13]. Because sex changes in both directions can be manipulated artificially and are reproducible in small aquariums, *T. okinawae* represents an excellent model for exploring its regulation during the initial phase at a genetic and molecular level.

To establish a transcriptomic signature associated with rapid sex change, RNA-sequencing was performed using the brains of *T. okinawae* and the resulting data were used for *de novo* assembly. We selected fish that had only undergone behavioural sex change in each direction, but not gonadal sex change, by artificially replacing individuals in an aquarium. Gene expression profiles were compared between fish that had behaviorally changed sex with those of stable males and females who did not undergo sex change for greater than or equal to one month in the laboratory. The transcriptomic changes were identified in the brain associated with the rapid changes occurring in response to social stimuli. Candidate genes implicated in rapid sex change were identified, which provide insight into the regulation of rapid sex change and the sexual plasticity of teleosts.

## 2. Material and methods

### 2.1. Sample collection and experimental operation

**Figure 1.**
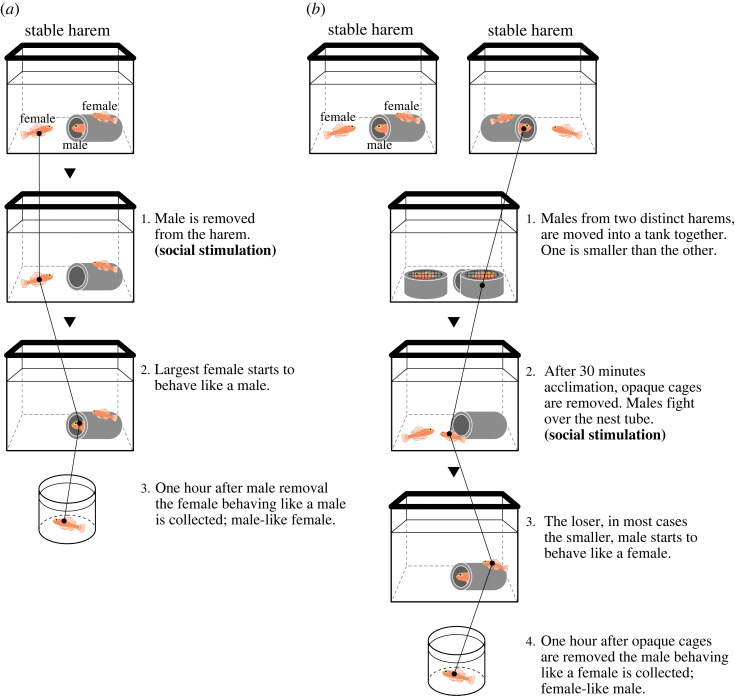
The sampling method of male-like females and female-like males. (*a*) The connected line consistently represents the largest female in the panels. The largest female behaving like a male, e.g. clean-up in the nest, territorial behaviour and courtship behaviour, was sampled as a male-like female. (*b*) The connected line consistently represents a smaller male in the panels. The losing male exhibiting subordinate behaviour, e.g. accepting courtship behaviour from the male, being expelled from the territory and keeping a distance from the nest tube, was considered a female-like male.

Fish were collected from a wild population by scuba diving in Makurazaki, Kagoshima Prefecture, Japan, in May 2016, and transported to the laboratory. To form a stable polygynous harem, we inserted a polyvinyl chloride tube as a nest site in each aquarium (60 cm width, 36 cm height and 30 cm depth) and maintained the harem for more than two weeks. Brains were harvested from males and females from stable harems as well as from behaviorally sex-changed males and females (five individuals per condition) 1 h after manipulation. We defined females behaving like males, i.e. showing territorial and nest-cleaning behaviour, as male-like females, while males behaving like females, i.e. showing subordinate behaviour and leaving the nest tube, as female-like males, 1 h after artificial manipulation (figure 1). Male-like females were obtained artificially by removing a dominant male from the harem. This is a stimulus for female-to-male sex change as the largest female in the harem adopts typical male behaviour. We selected this female one hour after the removal of the dominant male, and it was designated a male-like female (figure 1*a*). Meanwhile, female-like males were obtained through artificial encounters of males from two distinct harems. The males from two different tanks were moved to an experimental tank and acclimated for 30 min. During this time, each male was placed in an opaque cage (made of plastic netting and a thick PVC tube), so that they could not recognize one another. After removing the opaque cages, the males began to fight with one another over the PVC tube nest, which is a stimulus for a male-to-female sex change. The dominant male continued to behave as a male, whereas the loser began to exhibit typical female behaviour. We selected the losing male 1 h after the removal of the opaque cages and this male was designated a female-like male (figure 1*b*). The gonadal sex state of each fish was confirmed by urogenital papillae structure, which depends on gonadal sexes and manifests as a long tapered posterior in males and a bulbous appearance in females [12,14,15]. The animal experiments were approved by the committee of Tohoku University (Permit number: 2017LsA-023). The fish were euthanized with an overdose of ethyl 3-aminobenzoate methanesulfonate (MS-222; Sigma-Aldrich, St. Louis, MO; 100 mg l^−1^), their brains were immediately excised and placed into tubes containing *RNA*later stabilization solution (Thermo Fisher Scientific) within 30 min after taking the fish from the aquarium. The samples were stored at −80°C until RNA extraction.

### 2.2. RNA extraction and *de novo* assembly

Total RNA was extracted and purified from whole brain using a Maxwell 16LEV simply RNA tissue kit (Promega) based on the manufacturer's instructions. RNA was extracted from each of five females, males, male-like females and female-like males. In total, 20 RNA libraries were prepared using the NEBNext Ultra RNA Library Prep Kit for Illumina, and 150 bp paired-end reads were generated on the Illumina HiSeq 4000 platform. The quality of the sequencing reads were assessed using FastQC v0.11.7 [16] and low-quality reads and bases (Q < 20) were trimmed and discarded using Trimmomatic v0.36 [17]. *De novo* assembly was done using high-quality reads of all 20 samples by Trinity v2.6.5 using default parameters [18]). Subsequently, protein-coding sequences were predicted with TransDecoder v5.1.0 (https://github.com/TransDecoder/TransDecoder) using default parameters. To remove redundancy from protein-coding sequences, CD-hit v4.6.8 (cd-hit-est) was used with a sequence identity threshold of -c 0.95 19]. The comprehensiveness of the transcriptome dataset was assessed using the Benchmarking Universal Single-Copy Ortholog (v3) with the Actinopterygii dataset [20,21]. To search for orthologues and perform functional annotation, estimated protein-coding sequences were analysed by eggNOG-mapper v2 [22].

### 2.3. Gene expression analysis and gene ontology enrichment

**Figure 2.**
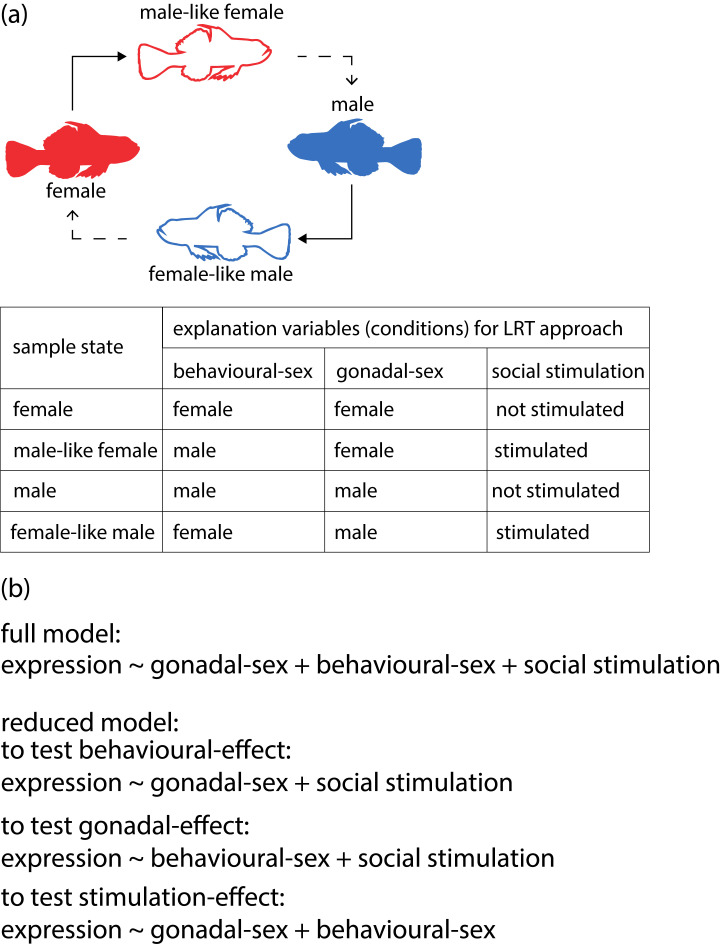
Overview of the LRT approach by each test using DEseq2. (*a*) Definition of explanation variables of the LRT in the present analysis. (*b*) The full model and reduced models used for each LRT.

RSEM v1.3.0 [23] with bowtie2 v2.3.5 [24] was used to map back the reads and calculate the gene-level expression abundance for each sample using high-quality reads with default parameters. Pairwise comparisons of gene expression levels of Actinopterygii orthologous genes estimated by eggNOG-mapper v2 for stable females versus males, females versus male-like females and males versus female-like males were done using the Wald test in DEseq2 v1.36.0 [25]. Genes were filtered out with very low expression (sum of expected count <10). Genes with a false discovering rate (FDR) less than 0.05 were considered differentially expressed genes (DEGs). As another approach, to detect genes that were associated with changes in behaviour, gonadal sex and social stimulation, a likelihood-ratio test (LRT) was performed using DEseq2 [25]. We established the full model consisting of explanatory variables, i.e. behavioural sex (female/female-like male or male/male-like female), gonadal sex (female/male-like female or male/female-like male) and social stimulation (female/male or female-like male/male-like female; figure 2). Then, LRTs were performed between the full model and each single model reducing a variable to detect the effect of each variable on the gene expression (figure 2*b*). If LRT using a reduced model focusing on behavioural sex detects significant differences of the likelihood for a given gene, the gene is assumed to be a behavioural sex-associated gene. Using this approach, we addressed only consistent gene expression changes associated with each variable (e.g. concordant upregulation in male-like females and female-like males by social stimulation). The genes detected from these analyses were used for gene ontology (GO) enrichment analysis based on GO annotations from eggNOG-mapper v2 using the GOseq v1.48.0 [26]. The FDR was calculated using overrepresented *p-*values by the Benjamini–Hochberg method [27] and GO terms with an FDR less than 0.05 were reported as significantly enriched GO terms. All statistical analyses were conducted using R 4.2.1 software (https://www.r-project.org/).

## 3. Results

*De novo* assembly and filtering resulted in 177 631 contigs coding for putative proteins (electronic supplementary material, table S1). An assessment of the completeness of the transcriptome assembly indicated that 82.1% (56.3% complete and 25.8% fragmented) single-copy orthologues were present, whereas 17.9% were missing. The putative proteins coded in the contigs were subjected to eggNOG-mapper v2 and annotated by taxonomic classification (electronic supplementary material, figure S1). Although most were classified into Actinopterygii orthologues (77 703 out of 129 505 proteins), the remainder were classified into orthologues of a different or uncertain taxon. The genes coding for Actinopterygii orthologue proteins included 29 762 putative protein-coding genes expressed in the brain of *T. okinawae*, which we used for subsequent analyses.

**Figure 3.**
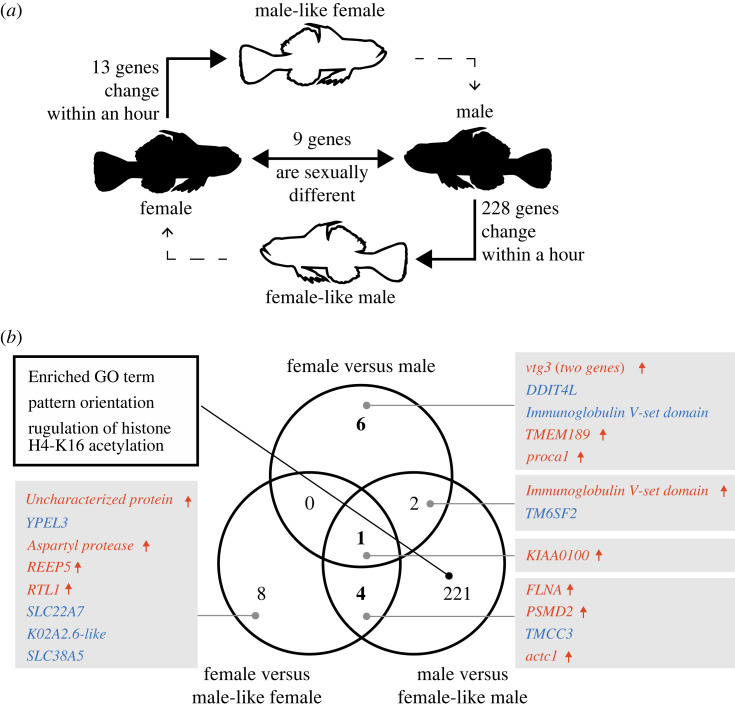
DEGs detected by pairwise comparisons. (*a*) Schematic illustration and the number of DEGs between female versus male, female versus male-like female and male versus female-like male, respectively, are shown. The solid lines represent sexual differences and behavioural sex change focused on in this study, while dotted lines represent a gradual change after that, i.e. gonadal sex change. (*b*) Venn diagram of a differentially expressed gene detected by three independent comparisons. The gene names next to the diagram in grey rectangles were obtained from eggNOG2-mapper annotation. The gene name colours, red and blue, represent upregulated and downregulated in female, respectively. The small arrows next to the gene name show upregulation in females.

The gene expression profiles revealed an unclear separation among the four groups (electronic supplementary material, figure S2). A pairwise comparison between stable females versus males revealed 6 female-biased and 3 male-biased DEGs (figure 3*a* and electronic supplementary material, table S2). We also identified 13 DEGs between females versus male-like females, and 8 female-biased and 5 male-like female-biased genes (figure 3*a* and electronic supplementary material, table S3). In contrast to the other two comparisons, we identified 228 DEGs between males versus female-like males and 84 of these were male-biased and 144 were female-like male-biased genes (figure 3*a*; electronic supplementary material, table S4). These DEGs for males versus female-like males exhibited a significant enrichment in ‘pattern orientation' and ‘regulation of histone H4-K16 acetylation', whereas no significant enrichment was observed for other comparisons (figure 3*b* and table 1; electronic supplementary material, tables S5–S7). Overlap of the DEGs from the three comparisons indicated that most of the genes were independently detected by each comparison (figure 3*b*). The intersection of the three comparisons revealed only one gene, which is *KIAA0100* orthologue. With respect to genes immediately controlled by social stimulation, four DEGs were detected by pairwise comparisons between females versus male-like females and males versus female-like males (figure 3*b*).

**Table 1.** Results of gene ontology enrichment analysis for pairwise comparison and LRT.

approach	comparison or used model	GO term	description	FDR
pairwise comparison	female versus male	—	—	—
	female versus male-like female	—	—	—
	male versus female-like male	GO:0007633	pattern orientation	0.025
		GO:2000618	regulation of histone H4-K16 acetylation	0.031
LRT	behavioural sex	GO:0040012	regulation of locomotion	0.037
	gonadal sex	—	—	—
	stimulated	GO:0007633	pattern orientation	0.011
		GO:2000618	regulation of histone H4-K16 acetylation	0.011
		GO:0097484	dendrite extension	0.027
		GO:2000620	positive regulation of histone H4-K16 acetylation	0.027
		GO:0098582	innate vocalization behaviour	0.032

**Figure 4.**
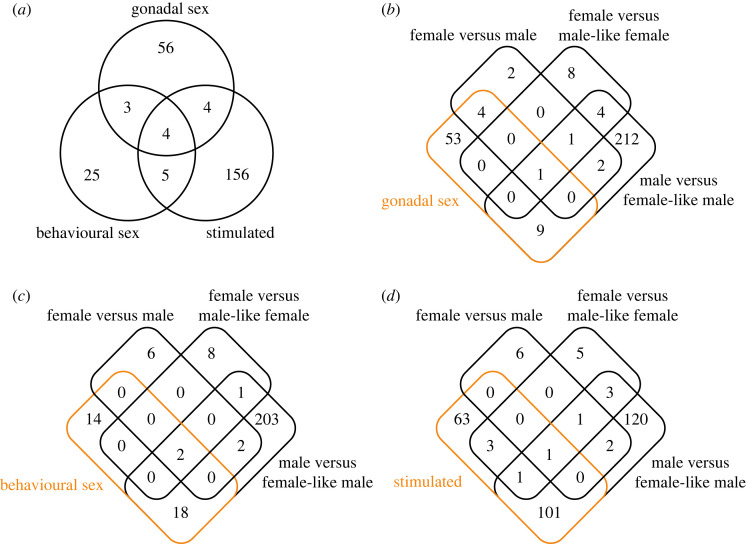
Venn diagram of the genes detected by the LRT approach. (*a*) Overlaps of the genes detected using each reduced model are shown. (*b–d*) Overlaps between the DEGs in the pairwise comparisons and the genes detected using reduced models focusing on gonadal sex, behavioural sex and stimulated, respectively, are shown.

To identify genes that consistently changed their expression based on sexual and social status in the brain, we conducted LRT to compare the full model with the reduced model (figures 2 and 4). The LRT using reduced models for each condition identified 36, 66 and 168 genes associated with behavioural sex, gonadal sex and social stimulation, respectively (figure 4; electronic supplementary material, figure S3 and tables S8–S10); however, the genes detected by each test showed little overlap (figure 4*a*). Using this approach, several genes exhibited intermediate, but significant expression changes, which were not detected by pairwise comparisons. For the gonadal sex-associated genes, 14 (20.9%) were also detected by pairwise comparisons and the other 53 were only detected by the LRT (figure 4*b*). By contrast, 23 (62.2%) and 106 (62.7%) genes were also identified by pairwise comparisons for the genes identified as behavioural sex-associated or social stimulation-associated, respectively, and most of the overlapped genes represented DEGs between males versus female-like males (figure 4*c,d*). GO enrichment analyses were performed and several overrepresented GO terms were identified, except for gonadal sex-associated genes (table 1 and electronic supplementary material, tables S11–S13). The behavioural sex-associated genes were significantly enriched in ‘regulation of locomotion’. The social stimulation-associated genes were significantly enriched in several GO terms including ‘pattern orientation' and ‘regulation of histone H4-K16 acetylation' (table 1).

## 4. Discussion

The transcriptomic changes occurring in the brain during the early phase of sex change are understudied. To our knowledge, previous studies using hermaphroditic species have primarily focused on changes in the gonads or the brain within a few days after the initiation of sex changes [28–30], except for recent work [31]. Previous studies using gonochoristic [32,33] and hermaphrodite fish [28–31,34] revealed few DEGs in the brain between the sexes. Our analysis also identified a small number of DEGs, which is consistent with the previous studies. Although the asymmetric treatment inducing sex change may affect the number of detected genes, especially through stress during transferring and fighting, we found several genes significantly associated with behavioural changes and social stimulation. In the present study, we could not exclude the effect of byproducts from artificial operations on gene expression, such as a winner effect following territorial conflicts and exhaustion after a fight. However, our analysis provides a unique dataset to examine the regulation of rapid responses in the brain during sex changes as well as a practical model system.

The *KIAA0100* orthologue, which is a lipid transporter, known as *BLTP2* or *BCOX1* in humans, exhibited sexually dimorphic expression. The expression levels were changed oppositely depending on the direction of the sex change (i.e. consistent with behavioural sex) (figure 3*b*). Although the information on other organisms is limited, the *KIAA0100* orthologue in *Drosophila melanogaster*, *hobbit*, regulates neuroendocrine exocytosis and its mutation results in a marked reduction in body size, because it fails to promote insulin secretion [35,36]. Because body size is an important factor for sex change of *T. okinawae* [12], the *KIAA0100* orthologue may be associated with sex change through body size and growth regulation*. Hobbit* is required for the maintenance of endocrine signalling [35], and therefore the orthologue of *KIAA0100* is possibly associated with neuroendocrine regulation during the sex change in the brain of *T. okinawae*.

Although only a few DEGs were identified in the females versus males comparison, several notable differences were detected in the gonadal sex-associated genes by the LRT. For example, the orthologues of BPI fold containing family C (*BPIFC*) were highly expressed in the brains of fish with female gonads (i.e. gonads of female and male-like female) in *T. okinawae* (electronic supplementary material, table S9). A previous study using Zebrafish revealed that this gene modulates the expression of *kiss2*, an orthologous gene regulating reproductive development through GnRH neuronal activity in mammals [37]. Interestingly, kisspeptin may have other neuroendocrinal functions on social and sexual behaviour by regulating neurons that express isotocin and vasotocin, although it is not a direct regulator of reproduction through GnRH neurons in several fish species [38–40]. Because these neurotransmitters may control behavioural sex change in a social context [11,31], regulatory changes in these factors may also play an important role in *T. okinawae*.

In the pairwise comparison between males versus female-like males, genes associated with the regulation of histone H4-K16 acetylation were enriched in the DEGs. Moreover, Autism Susceptibility Candidate 2 (*AUTS2*) and Sirtuin 1 (*SIRT1*) were identified as social stimulation-associated genes by the LRT. This suggests that these genes are regulated in the same manner by social stimulation, regardless of sex change direction (electronic supplementary material, table S10). *AUTS2* is a component of the polycomb group multiprotein PRC1-like complex, which activates gene transcription by recruiting histone acetyltransferase. It also plays various roles in neuronal migration and neuritogenesis [41,42]. *SIRT1* is a deacetylase that acts on histones and is abundantly expressed in the brain [43]. Histone acetylation in the brain is involved in social behaviour, including pair bonding in prairie voles and caste-related behaviour in ants [44,45], and is suggested to play crucial roles in the gonadal sex change of hermaphrodite fish [31,46]. In mammals, *SIRT1* epigenetically regulates *kiss1* expression and controls pubertal timing depending on environmental conditions (i.e. obesity and nutrition) [43]. *SIRT1* and *AUTS2* may regulate the chromatin state in kisspeptin neurons or other neurons through social stimulation and sex change in *T. okinawae*. From this analysis, three *AUTS2* orthologues were detected as both stimulation-associated genes and DEGs between male versus female-like male. Enrichment in GO, including ‘pattern orientation', ‘innate vocalization behaviour' and ‘dendrite extension’, largely relies on these orthologues. *AUTS2* is associated with various neuropathological conditions in humans and head size and neurodevelopment function in zebrafish [41,47]. Therefore, the expression shift may contribute to the transition of the neurological state associated with sex change in *T. okinawae*.

The orthologue of lysine demethylase 3B (*KDM3B*), which is lysine 9 of histone H3 (H3K9) demethylase, was identified as a social stimulation-associated gene by the LRT method (electronic supplementary material, table S10). The methylation of H3K9 and H3K4 are hallmarks of transcriptionally suppressed and activated chromatin. *KDM3B* knock-out XY mice were frequently sex-reversed [48]. A previous study of *KDM3B* knock-out mice revealed that circulating levels of 17β-oestradiol increased and impaired male-specific sexual behaviour [30]. Several H3K9 demethylases are highly expressed in the brain of the androdioecious killifish, *Kryptolebias marmoratus*, and exhibit significant sexually biased expression [49]. Based on these findings, *KDM3B* expression in the brain may respond to social stimulation and activate the genes associated with sexual development or behaviour in teleosts.

Our analysis proposed various genes, including novel candidates, associated with the initiation of sex changes in the brain. Future research requires a cautious interpretation of the function of these genes since some gene expressions in the brain are highly localized and therefore might be offset due to using the whole brain. The transcriptome dataset generated here provides potential targets for screening and sheds light on the initiation mechanism of sex changes in fish.

## Data Availability

The *de novo* transcriptome assembly, annotation of the transcriptome, RSEM outputs and the code are deposited in the Dryad Digital Repository (https://doi.org/10.5061/dryad.vdncjsz0r) [50]. Raw sequence reads can be found in the SRA database under BioProject ID: PRJDB15183. Supplementary material is available online [51].
